# Bioinformatic Exploration of the Targets of Xylem Sap miRNAs in Maize under Cadmium Stress

**DOI:** 10.3390/ijms20061474

**Published:** 2019-03-23

**Authors:** Baoxiang Wang, Dan Cheng, Ziyan Chen, Manman Zhang, Guoqiang Zhang, Mingyi Jiang, Mingpu Tan

**Affiliations:** 1 Lianyungang Institute of Agricultural Sciences in Jiangsu Xuhuai Region, Jiangsu Academy of Agricultural Sciences, Lianyungang 222000, China; wbxrice@163.com; 2 National Key Laboratory of Crop Genetics and Germplasm Enhancement, College of Life Sciences, Nanjing Agricultural University, Nanjing 210095, China; softcheng@njau.edu.cn (D.C.); 2018816133@njau.edu.cn (Z.C.); 2018116022@njau.edu.cn (M.Z.); 2015116027@njau.edu.cn (G.Z.); myjiang@njau.edu.cn (M.J.)

**Keywords:** xylem sap, miRNAs, target gene, cadmium, maize

## Abstract

Cadmium (Cd) has the potential to be chronically toxic to humans through contaminated crop products. MicroRNAs (miRNAs) can move systemically in plants. To investigate the roles of long-distance moving xylem miRNAs in regulating maize response to Cd stress, three xylem sap small RNA (sRNA) libraries were constructed for high-throughput sequencing to identify potential mobile miRNAs in Cd-stressed maize seedlings and their putative targets in maize transcriptomes. In total, about 199 miRNAs (20–22 nucleotides) were identified in xylem sap from maize seedlings, including 97 newly discovered miRNAs and 102 known miRNAs. Among them, 10 miRNAs showed differential expression in xylem sap after 1 h of Cd treatment. Two miRNAs target prediction tools, psRNAtarget (reporting the inhibition pattern of cleavage) and DPMIND (discovering Plant MiRNA-Target Interaction with degradome evidence), were used in combination to identify, via bioinformatics, the targets of 199 significantly expressed miRNAs in maize xylem sap. The integrative results of these two bioinformatic tools suggested that 27 xylem sap miRNAs inhibit 34 genes through cleavage with degradome evidence. Moreover, nearly 300 other genes were also the potential miRNAs cleavable targets without available degradome data support, and the majority of them were enriched in abiotic stress response, cell signaling, transcription regulation, as well as metal handling. These approaches and results not only enhanced our understanding of the Cd-responsive long-distance transported miRNAs from the view of xylem sap, but also provided novel insights for predicting the molecular genetic mechanisms mediated by miRNAs.

## 1. Introduction

Heavy metal accumulation in soils is of concern in agricultural production due to the adverse effects on food safety. Cadmium (Cd) is a non-essential element for plants, however it can be absorbed by the roots from the soil and transported to the aboveground parts; thus, it can not only affect the growth and the subsequent productivity of crops, but can also pose a great threat to human health because of its accumulation in the consumable parts of food crops [1,2,3,4]. MicroRNAs (miRNAs) are the most studied 20- to 22-nucleotide non-protein coding RNAs and are at the heart of regulating gene expression in multiple developmental and signaling pathways [5,6,7]. MiRNAs are hypersensitive to different heavy metals, such as Cd, aluminum, and lead in some crop plants (including rice, maize, oilseed rape, and radishes), and mounting evidence has revealed that miRNAs and their targets weaved networks play important regulatory roles in plant adaptation to different heavy metal stresses [4,8,9,10,11].

In plants, one of the most fascinating aspects of RNA silencing is its mobile nature, and the movement of small RNA (sRNA) molecules can “non-cell-autonomously” orchestrate developmental and stress responses [12,13]. Results obtained with grafting techniques and transient expression systems have shown that sequence-specific short interfering RNAs with a size of 21–24 nucleotides travel to distant organs [14,15]. Phloem exudates contain diverse miRNAs and at least two of them, *miR395* and *miR399*, involved in responses to nutrient availability, are transmitted through grafts, indicating long-distance movement [12,16,17]. Similarly, siRNA signals produced in source or sink tissues move from cell-to-cell and travel long distances via the phloem to apical tissues [18]. Though the long-distance transport of sRNAs was intensively investigated in phloem, small RNAs have also been isolated from the developing xylem of *Populus* stems, and a majority of these miRNAs have been predicted to target developmental- and stress/defense-related genes, including those associated with the biosynthesis of cell wall metabolites [19]. Arabidopsis *miR857*, specifically expressed in the vascular tissues of seedlings, is involved in regulating lignin content and consequently morphogenesis of the secondary xylem in by regulating the expression of its target gene *LACCASE7* [20].

MiRNAs negatively regulate their target gene expression at transcriptional and post-transcriptional levels by regulating both messenger RNA (mRNA) degradation and translational inhibition based on miRNA/target sequence complementarity [6]. High-throughput degradome sequencing has been successfully established and adapted to validate miRNA splicing targets in a variety of plant species, such as hyperaccumulator *Sedum alfredii* [21], *Populus* [22], rice [23,24], soybean [25], canola [5], and maize [26]. Recently, an integrated web-based tool, DPMIND (Degradome-based Plant MiRNA-Target Interaction and Network Database), was developed to scan sRNA targets in multiple plant species [27].

To thoroughly predict the roles of long-distance moving Cd-responsive maize miRNAs, three xylem sap sRNA libraries of Cd-stressed maize were constructed for high-throughput sequencing. Then, an integrative bioinformatic approach composed of psRNATarget and DPMIND was employed to predict potential targets of Cd-responsive xylem miRNAs. Intriguingly, 34 high-confidence cleavable targets for 27 xylem sap miRNAs were identified. Moreover, nearly 300 other genes were also the potential miRNAs cleavable targets, and the majority of them were enriched in abiotic stress response, cell signaling, transcription regulation, as well as metal handling, chelation, and storage. This investigation, therefore, would provide aid to elucidate the molecular genetic mechanisms underlying plant responses to Cd stress from the aspect of mobile miRNAs.

## 2. Results

### 2.1. High-Throughput Sequencing of sRNAs in Maize Xylem Sap

To investigate the differences in maize xylem sap sRNA profiles after Cd treatment, we collected xylem sap samples from Cd-treated seedlings, and three sRNA libraries (including two control samples) for sequencing were generated from the maize xylem sap.

We obtained about 2.7, 3.8, and 3.8 M total reads, represented by 0.96, 1.25, and 1.39 M unique sRNA reads, respectively, from the untreated 0 h (C0), untreated 1 h (C1), and Cd-treated 1 h (Cd1) libraries of xylem sap collected at the indicated time-point and treatment. The length distribution of reads showed that the majority of the reads were 20–24 nt in size, which was within the typical size range for Dicer-derived products [28].

**Figure 1:**
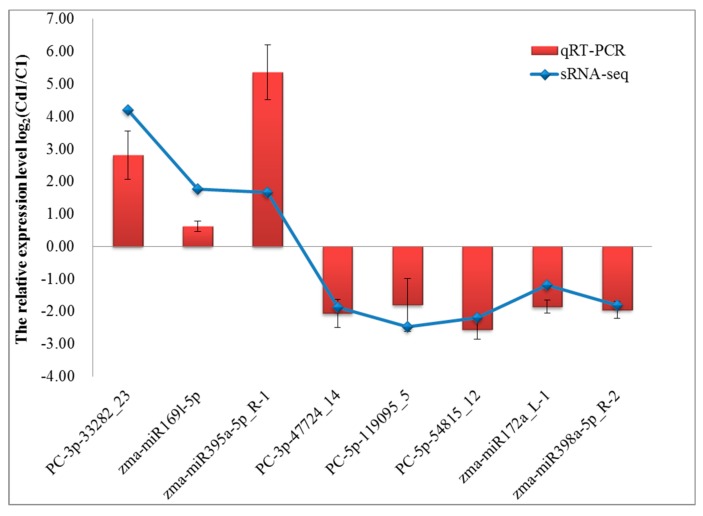
Validation and comparison of the relative expression pattern of the selected eight small RNAs (sRNAs) between qRT–PCR and high-throughput small RNA sequencing.

To confirm the expression of sRNAs identified by deep sequencing, eight sRNAs (lengths of 19–25 nt) were randomly selected for quantitative real-time RT-PCR (qRT-PCR) analysis, and these contained three miRNAs (*PC-3p-33282_23*, *zma-miR169l-5p*, and *zma-miR395a-5p_R-1*) with lengths of 20–22 nt (Supplementary Table S1). The comparison indicated that the expression patterns of Cd-responsive sRNAs from high-throughput sequencing and qRT-qPCR exhibited a good concordance (Figure 1, Supplementary Table S1), implying the reliability of the sRNA-seq profiling data for the following analysis.

The expression levels of sRNAs were compared between 1 h of Cd treatment (Cd1) and the control C1 samples. Data of qRT-qPCR are means ± SD from three independent biological replicates.

### 2.2. Identification of Xylem Sap Cd-Responsive miRNAs

After length filtration of the sequenced sRNAs, about 199 miRNAs (20–22 nt) were identified in xylem sap from Cd-treated or -untreated maize, including 97 newly discovered and 102 known miRNAs, which were homologous to the sequences in miRBase (Supplementary Table S2). Based on the number of reads (>10 at least in one sample) and MFEI (≥0.85) [29,30], about 20 new high-confidence miRNAs with relatively high expression levels were identified in the three samples (Table 1).

MiRNAs detected in C1 and Cd1 libraries were used for differential expression analysis using the stringent criteria (|log_2_Ratio| ≥1, *p* ≤ 0.05). Finally, 10 miRNAs showed differential expression in xylem sap after 1 h of Cd treatment (Table 2). Among them, the expressions of three newly identified miRNAs (*PC-3p-10246_108*, *PC-3p-33282_23*, and *PC-3p-65413_10*) was significantly regulated by Cd exposure (*p* ≤ 0.01).

Regarding the 10 Cd-modulated miRNAs, two highly expressed miRNAs (*zma-miR169l-5p*, *zma-miR398a-3p*), and two new miRNAs (*PC-3p-10246_108* and *PC-3p-33282_23*) were upregulated by Cd exposure; Cd significantly negatively regulated the remaining miRNAs (Table 2).

### 2.3. Target Predictions of Xylem Sap miRNAs

To better understand the biological functions of long-distance transported miRNAs, 199 significantly expressed miRNAs (*p* ≤ 0.05, in at least one dataset) from maize xylem sap were identified for target scanning (Supplementary File S1). The putative target sites in maize cDNAs were predicted using two plant sRNA target prediction tools (psRNAtarget and PsRobot).

With the application of psRNAtarget using the inhibition pattern of ‘Cleavage’, we identified a total of 2184 transcripts from 1436 maize genes, to be the targets of 196 xylem sap miRNAs (Supplementary Table S3). Using PsRobot, we obtained a total of 2514 transcripts from 1774 maize genes to be targets of 172 xylem sap miRNAs (Supplementary Table S4). Through the integration, we identified a total of 493 transcripts from 332 genes as the cleavable targets of 115 miRNAs in the intersection of results from psRNAtarget and PsRobot.

### 2.4. The Function Classification of the Predicted miRNAs Targets

To gain insights into the functionality of the miRNA targets, all of these 493 transcripts were functionally grouped by agriGO [31] and visualized in the candidate pathway networks with MapMan software [32].

**Figure 2:**
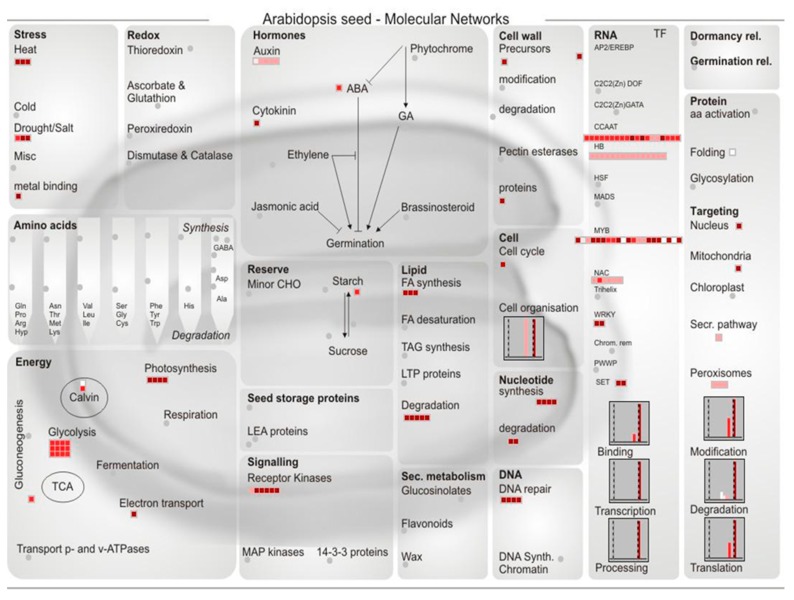
Global view of metabolic pathway-embedded targets the predicted cleavable targets of maize xylem miRNAs.

Among the genes within the ‘TF’ group, 11 members of MYB family, two WRKYs, and one AP2-EREBP as well as one bHLH-transcription factor were all the targets of *miR159* family members, whereas five NACs and six Homeobox-transcription factors were targeted by *miR164s* and *miR166s*, respectively (Table 3, Figure 2, Supplementary Table S3).

With regard to the targets mapped to “Secondary metabolism” category, nine laccases were exclusively coupled to *MIR397* family members. With regard to ‘Abiotic stress’ response, five genes (including two DNAJ proteins and one ERD ortholog) were targeted by five miRNAs individually. Similarly, within the ‘metal handling, chelation, and storage’ group, two major facilitator proteins and one MATE efflux family protein were uniquely targeted by three miRNAs. However, ZM2G058032 (heavy-metal-associated domain protein) and ZM2G407032 (ABC transporter) were co-targeted by *miR399s* (Table 3, Figure 2).

The predicted cleavable targets inhibited by miRNAs were inputted into MapMan software (3.6.0RC1) for metabolic pathways analysis (using the framework of Arabidopsis seed-Molecular Networks). The colored boxes indicated the expectation score output by psRNAtarget, and the larger value was shown in deep red.

In addition, several genes located in the pathway network were the potential targets of novel miRNAs. Particularly in the ‘signaling’ category, three genes involved in Ca signaling (ZM2G107575, ZM2G312661, and ZM2G174315) and the protein kinase (ZM2G100454) were uniformly targeted by novel miRNAs (Table 3, Figure 2).

### 2.5. The miRNAs Cleavable Targets

The transcripts of 332 maize genes in the intersection of psRNAtarget and PsRobot output, which were the potential 115 miRNAs cleavable targets, were further evidenced by the maize degradome data. With the aid of the DPMIND webserver [27], which harbors the degradome sequencing data of maize anther and ears, we obtained 34 maize genes harbored target sites of 27 xylem sap miRNAs by combining the outputs of psRNAtarget, PsRobot, and DPMIND (Table 4, Supplementary Table S5).

Regarding these cleavable candidates, the majority of them were intensively studied, including 11 squamosa promoter binding proteins (SBPs), seven nuclear transcription factor Ys (NFY), and three auxin response transcription factors (ARF) [6]. After excluding these well-known targets of miRNAs, several genes were filtered out as fresh cleavable targets for these xylem sap miRNAs, including the common stress-responsive miRNAs and their targets [6].

Many miRNAs appear to function together via co-targeting to regulate functionally related genes or pathways, and vice versa [33]. For example, the myb74 transcription factor (ZM2G028054_T03) was co-modulated by *zma-miR159a-3p_R-1* and *zma-miR319a-3p_R+1*, while myb138 (ZM2G139688_T01) was specifically targeted by *zma-miR159a-3p_R-1* (Table 4). In contrast, two F-box proteins (ZM2G064954_T01 and ZM2G119650_T01) were both the targets of *zma-miR394a-5p*. Similarly, ZM2G155490_T01 and ZM2G304745_T01 (encoding LRR receptor-like kinase) were targeted by *zma-miR390a-5p* (Table 4).

## 3. Discussion

### 3.1. The Long-Distance Transport of miRNAs

Long-distance transport of signaling molecules, intensively investigated in phloem, is known to be a major component in plant growth regulation, as well as their adaptation to changing environmental conditions [34]. Although some studies have demonstrated the presence of numerous miRNAs in phloem tissues [35,36,37], so far, only three miRNAs (*miR399*, *miR395*, and *miR172*) have been shown to move long distances in plants [16,17,34,38].

However, xylem also plays an important role in the root-to-shoot signaling system [39]. Furthermore, the root-to-shoot Cd translocation process may be more complex than previously thought [40]. Regarding xylem, research on miRNAs is in its infancy. In this study, about 199 miRNAs were identified in Cd-treated or -untreated maize xylem sap, including 97 novel and 102 known maize miRNAs. Moreover, the three famous long-distance moving miRNAs (*miR399*, *miR395*, and *miR172*) were detected in maize xylem sap (Table 3, Supplementary Table S2). These findings suggested that these miRNAs are potential signal molecules that move systemically.

MATE transporters were involved in the cellular transport and detoxification of Cd [41]. Furthermore, the distribution of allocated transcripts (e.g., MATE) along the root-to-shoot axis was correlated with the siRNA signal spread in hetero-grafted Arabidopsis [42]. Here, we identified *zma-miR528a-5p* in xylem sap, and predicted ZM2G148937 (MATE family protein) as its potential cleavable target (Table 3).

Six highly conserved amg-miRNA families (*amg-miR166*, *amg-miR172*, *amg-miR168*, *amg-miR159*, *amg-miR394*, and *amg-miR156*) were viewed as potential regulatory sequences of secondary cell wall biosynthesis [43], and Populus *Pto-MIR156c* might play vital roles in the regulation of wood formation in trees [44]. Moreover, the knockdown of rice *MicroRNA166* confers drought resistance by causing leaf rolling and altering stem xylem development [5], and rice *miR166* also plays a critical role in Cd accumulation and tolerance [45]. In this study, the miRNAs variants of these six families and nine *miR166* isoforms with more than 30 reads in each of the three samples were identified in maize xylem sap (Supplementary Table S2).

### 3.2. The Potential Cleavable Targets of miRNAs in Xylem Sap

In plants, miRNAs and their targets show a pattern of near complementarity, suggesting that plant miRNAs likely act through endonucleolytic cleavage of target mRNAs [46]. In this study, 34 targets were predicted to be inhibited by miRNAs through cleavage with degradome evidence, and most of them were intensively studied targets of common stress-responsive miRNAs, such as NFY, ARFs, SBPs, and GRAS family transcription factors [6]. Concerning the cleavable candidates of xylem sap miRNAs, they were concentrated on the NFY-, ARF-, SBP-, and GRAS-transcription factors, which was targeted by *zma-miR169s*, *zma-miR160f-5p*, *zma-miR156s*, and *zma-miR171s*, respectively (Table 4).

It is of particular interest to note that three homeobox-transcription factors were the cleavable targets of *zma-miR166s*, and two of them were annotated as rolled leaf genes (Table 4), which was reminiscent of their rice ortholog OsHB4. Moreover, *miR166* plays a critical role in Cd tolerance as well as in drought resistance through regulation of its cleavable HD-Zip target gene *OsHB4* in rice [5,45]. Altogether, these results of *miR166*-mediated regulatory cascade strengthened the pivotal role *miR166*-*HB* couple in abiotic stress acclimation. Moreover, among the miRNA targets within the ‘TF’ group, members of other TF families (e.g., MYBs, WRKYs, NACs) were the specific targets of certain miRNA family members (Table 3, Figure 2, Supplementary Table S3). These TF-type miRNA targets might contribute to elucidate the complicated mechanism of Cd stress from the aspect of transcriptional network through unveiling transcription factor-regulated downstream target genes which were involved in Cd stress response.

In addition to these well-known targets of miRNAs, several genes were filtered out as novel cleavable targets for these xylem sap miRNAs, including the common stress-responsive miRNAs and their targets [6] (Table 4).

Many miRNAs appear to function together via co-targeting to regulate functionally related genes, and vice versa [33]. For example, the myb74 was co-modulated by *zma-miR159a-3p_R-1* and *zma-miR319a-3p_R+1*, whereas two F-box proteins were both the targets of *zma-miR394a-5p* (Table 4). These co-targeting paradigms indicated that individual miRNA variants have different functions according to their specific targets [33].

Besides the 34 maize genes as the cleavable candidates of miRNAs with degradome evidence from the limited degradome data (Table 4), nearly 300 other genes were also the potential miRNAs cleavable targets (Table 3, Supplementary Table S3). From a global view, many target genes were prone to be enriched in abiotic stress response, cell signaling, transcription regulation, as well as metal handling, chelation, and storage (Table 3, Figure 2). Using a degradome sequencing approach, leucine-rich repeat (LRR) protein, cation transporting ATPase, and Myb transcription factors, were found to be cleaved by miRNAs under heavy metal stress [25]. Furthermore, a few miRNA cleavable targets, including iron transporter and ABC transporter, were involved in plant responses to Cd stress [2,8]. MATE transporters were involved in the cellular transport and detoxification of Cd [41]. In this study, ZM2G148937 (MATE family protein) was predicted as the cleavable target of *zma-miR528a-5p* (Table 3). From another perspective, these targets in relation to the Cd stress acclimation highlighted the role of the corresponding miRNAs in regulating Cd stress response.

Intriguingly, these known cleavable targets of miRNAs were also identified in this investigation (Table 3), though we did not retrieve degradome evidence from the available public dataset.

miRNAs were regarded as a new target for genetically improving plant tolerance to certain stresses [6]. From the perspective of miRNA-target couple, the characterization of the miRNAs and the associated targets in responses to Cd exposure will provide a framework for understanding the molecular mechanism of heavy metal tolerance in plants. Thus, it would be interesting to determine the role of these long-transported miRNAs, and whether these xylem sap miRNAs are transported to leaves under heavy metal stress or other stresses. Future investigation on the final location of xylem sap miRNAs, which might be achieved through analyzing the difference between the exudates from the node incisions where ears or leaves separated and xylem sap below the node, together with the target identification through degradome and proteome or ribosome profiling of the detached ears or leaves, will aid to illustrate the effect of xylem sap miRNAs on their potential targets in their final destination.

## 4. Materials and Methods

### 4.1. Plant Materials and Cd Treatment

The seedlings of maize (*Zea mays* L. cv. Nongda 108; China) were cultivated in a hydroponic system in a growth chamber with a temperature of 22 °C (night) to 28 °C (day), photosynthetic active radiation of 200 μmol·m^−2^·s^−1^, and a 14/10-h day/night photoperiod. All hydroponic solutions were continuously aerated and renewed every three days. When the third leaves were fully expanded, the seedlings were transferred into fresh growing solutions containing 100 uM CdCl_2_, according to previous reports [2,3].

### 4.2. Sampling of Xylem Sap

The seedlings were separated into three groups—untreated 0 h (C0), untreated 1 h (C1), and Cd-treated 1 h (Cd1). For each group, 30 maize seedlings at the indicated timepoint/treatment were de-topped by cutting the stem with a razor blade just above the first internode, and the remaining parts without stem were used for xylem sap collection, according to previous reports with minor modifications [47,48,49,50,51]. Then, the cut surface was rinsed twice with distilled water and the liquid drawn in the first 5 min was discarded. Finally, the bleeding saps from 30 maize plants were harvested with 10 uL syringe for 1 h after cutting and mixed in a tube containing 1 mL Trizol as one sample replication, and single biological replication for each sample was used for sRNA sequencing.

### 4.3. Small RNA Library Preparation and Sequencing

Total RNA was extracted from C0, C1, and Cd1 xylem sap samples using Trizol reagent (Invitrogen, Carlsbad, CA, USA) following the manufacturer’s procedure. Approximately 1 µg of total RNA were used to prepare a small RNA library (single biological replication for each sample) according to protocol of TruSeq Small RNA Sample Prep Kits (Illumina, San Diego, CA, USA). Then, we performed the single-end sequencing (36 bp) on an Illumina Hiseq2500 at the LC-BIO (Hangzhou, China) following the vendor’s recommended protocol. The data was uploaded to NCBI/SRA with accession number SRP073229 (https://trace.ncbi.nlm.nih.gov/Traces/study/?acc=SRP073229), and contained miRNAs reads of C0, C1, and Cd1 xylem sap samples (single biological replication for each sample).

### 4.4. Small RNA Analysis

Data processing followed the procedures as described previously [23,24]. The raw reads were subjected to the Illumina pipeline filter (Solexa 0.3), and then the dataset was further processed with an in-house program, ACGT101-V4.2 (LC Sciences, Houston, TX, USA) to remove adapter dimers, junk, low complexity, common RNA families (rRNA, tRNA, snRNA, snoRNA), and repeats.

### 4.5. Identification of Known and Novel miRNAs

Unique sequences with lengths of 20–22 nucleotide [52] were mapped to monocot plant precursors in miRBase Release 22.1 (October 2018, mirbase.org) by BLAST search to identify known miRNAs and novel 3p- and 5p-derived miRNAs.

The unique sequences mapping to maize mature miRNAs in hairpin arms were identified as known miRNAs. The unique sequences mapping to the other arm of known maize precursor hairpin opposite to the annotated mature miRNA-containing arm were considered to be novel 5p- or 3p-derived miRNA candidates. The remaining sequences were mapped to other monocot precursors (with the exclusion of maize) in miRBase 20.0 by BLAST search, and the mapped pre-miRNAs were further BLASTed against the maize genomes (ftp://ftp.maizesequence.org/pub/maize/release-5b/assembly/ZmB73_RefGen_v2.tar.gz with genome annotation file ZmB73_5b_FGS.gff.gz) to determine their genomic locations [53].

The sequences unmapped to maize mature miRNAs or other monocot miRNAs precursors were further BLASTed against the maize genomes, and the hairpin RNA structures containing mappable sequences were predicted from the flank 120 nt sequences using RNAfold software (http://rna.tbi.univie.ac.at/cgi-bin/RNAfold.cgi).The criteria used to annotate potential miRNAs was as described previously [54,55,56,57]. Sequences that met the criteria for secondary structure prediction were then considered to be novel miRNA precursors [53,58,59]. Minimum free energy index (MFEI) was also taken into account for evaluating the confidence of novel miRNAs, with the expected value (≥0.85) [7,29,30]. Moreover, all of the aforementioned criteria must be fulfilled in at least two distinct sRNA-seq libraries [52].

### 4.6. Differential Expression Analysis of sRNAs Under Cd Stress

Data normalization followed the procedures as described in a previous study [60]. sRNA differential expression based on normalized deep-sequencing counts was analyzed by selectively using Fisher’s exact test and Chi-squared 2 × 2 test with the selection threshold of 0.05 [23,28,60].

To investigate the differentially expressed miRNAs between libraries, we compared the gene expression patterns of miRNAs in Cd1 and C1 library. Towards this purpose, we considered the following criteria: (1) *p*-value should be less than 0.05 (*p* ≤ 0.05) in at least one dataset; and (2) Log_2_ ratio of fold change between normalized counts of C1 and Cd1 libraries was greater than 1 or less than −1 [28,56,57,59,61].

### 4.7. The Prediction of miRNA Targets

After filtering, we get 199 significantly expressed miRNAs (*p* ≤ 0.05, in at least one of the three samples). Then, these xylem sap 199 miRNAs were used to interrogate maize annotated cDNAs sequences (Zea_mays.AGPv3.22, ftp://ftp.ensemblgenomes.org) preloaded in the psRNAtarget web server (plantgrn.noble.org/psRNATarget) [62] for predicting target sites as described previously [61], and the default criteria for target prediction in psRNAtarget website were used.

Prediction of miRNA target genes was also performed by the local version of psRobot_tar scripts in psRobot (omicslab.genetics.ac.cn/psRobot) [63] using the 199 miRNAs for scanning maize cDNAs sequences (http://ftp.maizesequence.org/release-5a/filtered-set/ZmB73_5b_FGS_cdna.fasta.gz) with default settings (moderate model of pre-set the target penalty score 2.5, thus alignments that meet the penalty score cutoff will be reported in the result).

To scan the cleavable targets of the identified known miRNAs and novel miRNAs, these miRNAs were uploaded to DPMIND (http://cbi.njau.edu.cn/DPMIND) [27] to locate the homologous miRNAs for the following degradome data query.

### 4.8. The Confirmation of sRNAs Expression by qRT-PCR

To confirm the expression of sRNAs identified by deep sequencing, eight sRNAs (lengths of 19–25 nt) were randomly selected for quantitative real-time RT-PCR (qRT-PCR) analysis (Supplementary Table S1), and these contained 3 identified miRNAs (with lengths of 20–22 nt) [7,57,61].

For determining the expression of sRNAs, about 2 μg RNAs were reverse-transcribed by miRcute miRNA First-Strand cDNA Synthesis kit (TIANGEN, Beijing, China). Transcript levels of mature sRNAs were measured by qRT-PCR using a DNA Engine Opticon 2 real-time PCR detection system (Bio-Rad, Hercules, CA, USA) with miRcute miRNA qPCR Detection kit (TIANGEN) according to the manufacturer’s instructions. Details of the primers used are listed in Supplementary Table S1. The maize *5S RNA* was used as the internal control for RNA template normalization [64]. All reactions were run in triplicate. The relative expression levels of *sRNAs* were calculated by the comparative threshold cycle (*Ct*) method. At least three independent biological replicates were used for each small RNA.

## Figures and Tables

**Table 1:** Novel MicroRNAs (miRNAs) with high-confidence in maize xylem sap.

miRNA	c0	C1	Cd1	Sequence (5′-3′)	Size	MFEI
cme-MIR156j-p3	27	22	52	GCTCACTTCTCTTTCTGTCAGT	22	0.90
sof-MIR156-p3	324	470	780	GCTCACTTCTCTCTCTGTCAGC	22	1.00
zma-MIR166n-p3	1179	856	835	GCTGTCGTCGACCGGAGATC	20	1.00
zma-MIR169g-p3	8	6	10	GGCGGTCTCCTTGGCTAGCC	20	1.00
zma-MIR171f-p3	30	49	41	TGATTGAGCCGTGCCAATATC	21	0.90
sbi-MIR171h-p3	18	19	10	TTGAGCCGCGTCAATATCTC	20	1.10
zma-MIR397b-p5	105	185	190	TTGAGCGCAGCGTTGATGAGC	21	0.90
PC-5p-37430_20	14	16	16	TCTCTTAAGGCTTGTTCGGA	20	0.90
PC-5p-27068_30	23	12	17	ACCGGAGGAGGTTAGAGGAGC	21	1.30
PC-5p-14301_71	41	76	65	GGTTTTAGCTTCAAGCCATCT	21	0.90
PC-5p-10912_100	30	52	52	CCGGAAATACCCAATATCTTG	21	1.00
PC-3p-7571_159	50	103	60	GGTGGCTTGTGGCTAAAACCA	21	0.90
PC-3p-65413_10	4	10	1	GCTTTAAGGGATCTGTTGGAGA	22	1.00
PC-3p-52974_13	12	8	13	AATGGTGCATTGACTTGGTC	20	1.10
PC-3p-49169_14	6	8	16	TTTGTCAATTTAAGAACTAAAA	22	1.80
PC-3p-37537_20	88	62	77	AATACTGAGCCGAATTGAAAT	21	1.10
PC-3p-33282_23	11	1	17	GCATCCATTCTTGGCTAAGTG	21	1.20
PC-3p-18761_49	21	46	43	GCCTGTATGCACTCTCGGTG	20	0.90
PC-3p-18578_50	17	22	20	TTTATGATATGTTACTCTACT	21	1.50
PC-3p-10246_108	65	7	44	CAGGCCTTCTTGGCTAAGCG	20	0.90
PC-3p-100706_6	8	8	15	GGAGCTGCAAACACTCTGGT	20	1.50
osa-MIR1430-p5	58	34	63	CTTAGCCAAGAATGGCTTGCCT	22	1.00

The numbers under c0, C1, and Cd1 represent the number of normalized miRNA reads. Novel miRNAs with high-confidence, meaning that the number of reads (>10 at least in one sample) and Minimum free energy index (MFEI ≥0.85), in xylem sap are listed here. c0 represents 0 h of untreated sample, C1 represents 1 h of untreated sample, and Cd1 represents 1 h of Cd-treated sample. Size indicated the length (nt) of miRNAs.

**Table 2:** Cadmium (Cd)-responsive 10 miRNAs in maize xylem sap.

miRNA	C1	Cd1	log_2_FC	chr	Strand	Start	End	hairpinLen
**Cd Upregulated**								
***PC-3p-10246_108***	7	44	2.73	chr3	+	229987606	229987776	167
*PC-3p-33282_23*	1	17	4.20	chr3	−	96704531	96704709	176
**zma-miR169l-5p**	31	107	1.77	chr1	+	298277019	298277107	87
zma-miR398a-3p	31	73	1.23	chr7	+	38540171	38540278	106
zma-miR398a-3p	31	73	1.23	chr2	+	169527758	169527897	138
**Cd downregulated**								
zma-miR164d-3p_R+1	6	1	−2.47	chr7	−	172723300	172723515	214
*PC-3p-74571_8*	9	4	−1.21	chr2	+	22503757	22503904	142
*PC-5p-167816_4*	5	1	−2.21	chr8	−	103189069	103189246	135
*PC-5p-395659_2*	5	1	−2.21	chr1	+	296265246	296265464	216
*PC-5p-76360_8*	5	1	−2.21	chr1	−	52464270	52464417	103
***PC-3p-65413_10***	10	1	−3.34	chr6	+	161474760	161474953	129

C1 and Cd1 mean the 1 h of Cd-untreated and -treated samples, respectively. The numbers under C1 and Cd1 represent the number of normalized miRNA reads (norm reads). Novel miRNAs are listed in italic font and Cd significantly regulated miRNAs are in bold font.

**Table 3:** The function classification of the predicted cleavable targets of maize xylem miRNAs.

	Gene IDs		Function	miRNA(s) ^$^
**Abiotic Stress**				
ZM2g053531			Wound-responsive protein	miR164s
ZM2G129218			DNAJ heat family protein	miR1432s
ZM2G134917			DNAJ homolog1 chaperone	miR395s
ZM2G456000			ERD, early-responsive to dehydration	miR396s
ZM2g042295			SAM-dependent methyltransferase	PC-5p-87289_7
**Phytohormone**				
ZM2G159399	ZM2G081406	AC207656.3_FG002	auxin response factor	miR160s
ZM2G078274			auxin response factor	miR6253s
ZM2g137451	ZM5g848945	ZM2G135978	auxin signaling F-box	miR393s
ZM2g095786			F-box protein FBX14	MIR159s
ZM2G019799			aldehyde oxidase3	PC-3p-172602_4
**Secondary Metabolism**				
ZM2G336337	ZM2G388587	ZM2G164467	laccase	MIR397s
ZM2G094375	ZM2G305526	ZM2G400390		
ZM2G072780	ZM2G072808	ZM2G447271		
ZM2G145029			isopentenyl pyrophosphate isomerase2	PC-3p-158678_4
**Metal Handling, Chelation, and Storage**				
ZM2G085301			Major facilitator superfamily protein	miR166s
ZM2G166976			Major facilitator superfamily protein	miR827s
ZM2G148937			MATE efflux family protein	miR528s
ZM2G058032			Heavy-metal-associated domain	miR399s
ZM2G407032			ABC transporter I family member 1	
ZM2G000039			SIT4 phosphatase-associated protein	miR171s
**Transcription Factors**				
ZM2G033356			bHLH-transcription factor, bHLH130	PC-3p-151371_4
AC193786.3_FG005			bHLH-transcription factor, Bhlh154	MIR159s
ZM2G027960			Zinc finger protein WIP6	
ZM2G399098			AP2-EREBP-transcription factor 124	
ZM2G048450	ZM2G111711		WRKY-transcription factor	
ZM2G093789	ZM2G416652	ZM2G167088	MYB transcription factor	
ZM2G423833	ZM2G075064	ZM2G376684		
ZM2G046443	ZM2G070523	ZM2G139688		
ZM2G004090	ZM2G028054		MYB transcription factor	miR159s, miR319s
ZM2G127490	ZM2G171781		MYB transcription factor	PC-5p-76360_8
ZM2G305856	ZM2G096358		MYB transcription factor	miR164s
ZM2G139700	ZM2G393433	ZM2G114850	NAC-transcription factor	
ZM2G063522	ZM2G146380			
ZM2G003509	ZM2G042250	ZM2G178102	Homeobox-transcription factor	miR166s
ZM2G469551	ZM2G109987	AC187157.4_FG005	Homeobox-transcription factor	
**Signaling**				
ZM2g012584			IQ-domain	miR164s
ZM2G104730			Calcium-transporting ATPase 9	miR169s
ZM2G107575			calcineurin B-like1	PC-3p-89447_7
ZM2G312661			Calcium-binding protein CML42	PC-3p-327923_2, PC-3p-100706_6
ZM2G174315			CaM-binding heat-shock protein	PC-3p-513669_2
ZM2G100454			Protein kinase	PC-5p-442461_2
ZM2G391794	ZM2G061447	ZM2G146346	LRR receptor-like kinase	MIR159s
ZM2G304745			LRR receptor-like kinase	miR390s
ZM2G145756	ZM2G082522		Protein kinase	miR167s

**^$^** label means miRNA family members, designated as miRs.

**Table 4:** The cleavable targets of maize xylem miRNAs.

miRNA	Target	psRNAtarget		DPMIND		Annotation (maizeGDB)
		Exp *	UPE	miR	Deg ^$^	
zma-miR394a-5p	ZM2G064954_T01	0	23.11	zma-miR394a-5p	2	LOC103636344 F-box only protein
	ZM2G119650_T01	0	22.97		1	LOC100193727, F-box domain
zma-miR393b-5p_R-1	ZM2G135978_T01	1	18.85	zma-miR393b-5p	1	transport inhibitor response 1-like
zma-miR390a-5p	ZM2G155490_T01	2	9.90	zma-miR390a-5p	1	GRMZM2G155490
	ZM2G304745_T01	1	21.54		1	LOC103648480 LRR receptor-like kinase
zma-miR827-5p_L+1	ZM2G044788_T01	2.5	20.66	zma-miR827-5p	1	LOC100274914
zma-miR160f-5p_1ss21GA	AC207656.3_FGT002	0	24.06	zma-miR160f-5p	1	arftf19, ARF-transcription factor
	ZM2G081406_T01	1	22.15		1	arftf15
	ZM2G159399_T01	0	21.94		2	arftf17
zma-miR159a-3p_R-1	ZM2G028054_T03	1.5	16.12	zma-miR159a-3p	1	myb74 transcription factor
zma-miR319a-3p_R+1		1	16.03	zma-miR319a-3p	2	
zma-miR159a-3p_R-1	ZM2G139688_T01	2	17.18	zma-miR159a-3p	3	myb138
gma-miR171m_1ss21AC	ZM2G098800_T01	0.5	23.32	zma-miR171m-3p	1	gras80 transcription factor
sbi-MIR171h-p3		0.5	23.32		1	
osa-MIR171a-p3		0	23.32	zma-miR171n-3p	1	
zma-MIR171f-p3		0.5	23.52	zma-miR171f-3p	3	
*zma-miR166a-3p*	ZM2G109987_T04	1	24.23	zma-miR166a-3p	2	rld1, rolled leaf, Homeobox
*osa-miR166a-3p_1ss21CT*	ZM2G042250_T04		23.53	zma-miR166c-3p		rld2
*zma-miR166l-3p*	ZM2G469551_T02		19.01	zma-miR166l-3p		hb69, Homeobox-transcription factor
*lus-miR169a_R-1*	ZM2G000686_T06	2	18.96	zma-miR169h	6	nfya1 nuclear transcription factor Y
*zma-miR169a-5p_R-1*	ZM2G040349_T01	2	18.79	zma-miR169a-5p	2	ca2p4, NFY/CCAAT-HAP2-transcription factor
*zma-miR169f-5p_R-1_1ss1TG*	ZM2G091964_T02	2.5	20.96	zma-miR169f-5p	7	ca2p16
*zma-miR169i-5p_R-1*	ZM2G038303_T01	2	16.87	zma-miR169i-5p	6	ca2p15
*zma-miR169l-5p*	ZM5G857944_T03	2.5	17.47	zma-miR169l-5p	6	ca2p13
*zma-miR169o-5p_R-1*	ZM5G853836_T01	2.5	20.94	zma-miR169o-5p	4	ca2p5
osa-miR169b_R+1	ZM5G857944_T03	2	17.47	zma-miR169c-5p	6	ca2p13
	ZM2G067624_T02	1	16.26		7	sbp29, squamosa promoter binding protein
*bdi-miR156b-5p_R+2*	ZM2G097275_T04		21.87	zma-miR156i-5p	1	sbp27
*osa-miR156a_R+1*	ZM2G113779_T01		14.23	zma-miR156a-5p	5	sbp13
*zma-miR156a-5p*	ZM2G126018_T01		18.45	zma-miR156a-5p	1	sbp23
*zma-miR156j-5p_R-1*	ZM2G126827_T01		22.70	zma-miR156j-5p	1	sbp12
*zma-miR156k-5p*	ZM2G156621_T01		22.70	zma-miR156k-5p	1	sbp31
*zma-miR529-5p*	ZM2G307588_T01		16.69	zma-miR529-5p	1	tsh4, tassel sheath4, SBP
	ZM2G371033_T01		18.52		3	sbp18
	ZM5G878561_T01		19.13		3	sbp22
	ZM2G460544_T01		20.77		1	ub3, unbranched3, SBP
zma-miR529-5p	ZM2G160917_T01	0.5	22.12	zma-miR529-5p	1	ub2, unbranched2

The miRNAs in italic and those maize transcripts between parallel lines mean each of the miRNAs can target each transcript successively. psRNAtarget output: Exp for Maximum expectation and the star (*) indicating the largest score of the corresponding miRNA-target combinations, unpaired energy (UPE) for maximum energy to unpaired target site; For DPMIND, miR represents the homolog of the queried miRNA by BLAST, and the dollar label (^$^) means the least number of the degradome datasets for the miR-target associations.

## Supplementary Materials

Supplementary materials can be found at https://www.mdpi.com/1422-0067/20/6/1474/s1.

## References

[1] Uraguchi S., Fujiwara T. (2012). Cadmium transport and tolerance in rice: Perspectives for reducing grain cadmium accumulation. Rice.

[2] Kulik A., Anielska-Mazur A., Bucholc M., Koen E., Szymanska K., Zmienko A., Krzywinska E., Wawer I., McLoughlin F., Ruszkowski D. (2012). SNF1-related protein kinases type 2 are involved in plant responses to cadmium stress. Plant Physiol..

[3] He F., Liu Q., Zheng L., Cui Y., Shen Z. (2015). RNA-seq analysis of rice roots reveals the involvement of post-transcriptional regulation in response to cadmium stress. Front. Plant Sci..

[4] Jian H., Yang B., Zhang A., Ma J., Ding Y., Chen Z., Li J., Xu X., Liu L. (2018). Genome-wide identification of MicroRNAs in response to cadmium stress in oilseed rape (*Brassica napus* L.) using high-throughput sequencing. Int. J. Mol. Sci..

[5] Zhang J., Zhang H., Srivastava A.K., Pan Y., Bai J., Fang J., Shi H., Zhu J.K. (2018). Knockdown of rice MicroRNA166 confers drought resistance by causing leaf rolling and altering stem xylem development. Plant Physiol..

[6] Zhang B. (2015). MicroRNA: A new target for improving plant tolerance to abiotic stress. J. Exp. Bot..

[7] Zhao P., Ding D., Zhang F., Zhao X., Xue Y., Li W., Fu Z., Li H., Tang J. (2015). Investigating the molecular genetic basis of heterosis for internode expansion in maize by microRNA transcriptomic deep sequencing. Funct. Integr. Genom..

[8] Xu L., Wang Y., Zhai L., Xu Y., Wang L., Zhu X., Gong Y., Yu R., Limera C., Liu L. (2013). Genome-wide identification and characterization of cadmium-responsive microRNAs and their target genes in radish (*Raphanus sativus* L.) roots. J. Exp. Bot..

[9] Kong X., Zhang M., Xu X., Li X., Li C., Ding Z. (2014). System analysis of microRNAs in the development and aluminium stress responses of the maize root system. Plant Biotechnol. J..

[10] Tang M., Mao D., Xu L., Li D., Song S., Chen C. (2014). Integrated analysis of miRNA and mRNA expression profiles in response to Cd exposure in rice seedlings. BMC Genom..

[11] Ding Y., Ye Y., Jiang Z., Wang Y., Zhu C. (2016). MicroRNA390 is involved in cadmium tolerance and accumulation in rice. Front. Plant Sci..

[12] Marin-Gonzalez E., Suarez-Lopez P. (2012). “And yet it moves”: Cell-to-cell and long-distance signaling by plant microRNAs. Plant Sci..

[13] Himber C., Dunoyer P. (2015). The tracking of intercellular small RNA movement. Methods Mol. Biol..

[14] Zhang W., Kollwig G., Stecyk E., Apelt F., Dirks R., Kragler F. (2014). Graft-transmissible movement of inverted-repeat-induced siRNA signals into flowers. Plant J..

[15] Khaldun A.B., Huang W., Lv H., Liao S., Zeng S., Wang Y. (2016). Comparative profiling of miRNAs and target gene identification in distant-grafting between tomato and lycium (*Goji berry*). Front. Plant Sci..

[16] Pant B.D., Buhtz A., Kehr J., Scheible W.R. (2008). MicroRNA399 is a long-distance signal for the regulation of plant phosphate homeostasis. Plant J..

[17] Buhtz A., Pieritz J., Springer F., Kehr J. (2010). Phloem small RNAs, nutrient stress responses, and systemic mobility. BMC Plant Biol..

[18] McGarry R.C., Kragler F. (2013). Phloem-mobile signals affecting flowers: Applications for crop breeding. Trends Plant Sci..

[19] Puzey J.R., Karger A., Axtell M., Kramer E.M. (2012). Deep annotation of *Populus trichocarpa* microRNAs from diverse tissue sets. PLoS ONE.

[20] Zhao Y., Lin S., Qiu Z., Cao D., Wen J., Deng X., Wang X., Lin J., Li X. (2015). MicroRNA857 Is involved in the regulation of secondary growth of vascular tissues in Arabidopsis. Plant Physiol..

[21] Han X., Yin H., Song X., Zhang Y., Liu M., Sang J., Jiang J., Li J., Zhuo R. (2016). Integration of small RNAs, degradome and transcriptome sequencing in hyperaccumulator *Sedum alfredii* uncovers a complex regulatory network and provides insights into cadmium phytoremediation. Plant Biotechnol. J..

[22] Lian C., Yao K., Duan H., Li Q., Liu C., Yin W., Xia X. (2018). Exploration of ABA responsive miRNAs reveals a new hormone signaling crosstalk pathway regulating root growth of *Populus euphratica*. Int. J. Mol. Sci..

[23] Li W., Jia Y., Liu F., Wang F., Fan F., Wang J., Zhu J., Xu Y., Zhong W., Yang J. (2019). Integration analysis of small RNA and degradome sequencing reveals MicroRNAs responsive to *Dickeya zeae* in resistant rice. Int. J. Mol. Sci..

[24] Li Y.F., Zheng Y., Addo-Quaye C., Zhang L., Saini A., Jagadeeswaran G., Axtell M.J., Zhang W., Sunkar R. (2010). Transcriptome-wide identification of microRNA targets in rice. Plant J..

[25] Zeng Q.Y., Yang C.Y., Ma Q.B., Li X.P., Dong W.W., Nian H. (2012). Identification of wild soybean miRNAs and their target genes responsive to aluminum stress. BMC Plant Biol..

[26] Liu H., Qin C., Chen Z., Zuo T., Yang X., Zhou H., Xu M., Cao S., Shen Y., Lin H. (2014). Identification of miRNAs and their target genes in developing maize ears by combined small RNA and degradome sequencing. BMC Genom..

[27] Fei Y., Wang R., Li H., Liu S., Zhang H., Huang J. (2018). DPMIND: Degradome-based plant miRNA-target interaction and network database. Bioinformatics.

[28] Yang J., Liu X., Xu B., Zhao N., Yang X., Zhang M. (2013). Identification of miRNAs and their targets using high-throughput sequencing and degradome analysis in cytoplasmic male-sterile and its maintainer fertile lines of *Brassica juncea*. BMC Genom..

[29] Zhang B.H., Pan X.P., Cox S.B., Cobb G.P., Anderson T.A. (2006). Evidence that miRNAs are different from other RNAs. Cell. Mol. Life Sci..

[30] Guzman F., Almerao M.P., Korbes A.P., Christoff A.P., Zanella C.M., Bered F., Margis R. (2013). Identification of potential miRNAs and their targets in *Vriesea carinata* (Poales, Bromeliaceae). Plant Sci..

[31] Tian T., Liu Y., Yan H., You Q., Yi X., Du Z., Xu W., Su Z. (2017). agriGO v2.0: A GO analysis toolkit for the agricultural community, 2017 update. Nucleic Acids Res..

[32] Usadel B., Poree F., Nagel A., Lohse M., Czedik-Eysenberg A., Stitt M. (2009). A guide to using MapMan to visualize and compare Omics data in plants: A case study in the crop species, Maize. Plant Cell Environ..

[33] Huang D., Koh C., Feurtado J.A., Tsang E.W., Cutler A.J. (2013). MicroRNAs and their putative targets in *Brassica napus* seed maturation. BMC Genom..

[34] Bhogale S., Mahajan A.S., Natarajan B., Rajabhoj M., Thulasiram H.V., Banerjee A.K. (2014). MicroRNA156: A potential graft-transmissible microRNA that modulates plant architecture and tuberization in *Solanum tuberosum* ssp. andigena. Plant Physiol..

[35] Yoo B.C., Kragler F., Varkonyi-Gasic E., Haywood V., Archer-Evans S., Lee Y.M., Lough T.J., Lucas W.J. (2004). A systemic small RNA signaling system in plants. Plant Cell.

[36] Buhtz A., Springer F., Chappell L., Baulcombe D.C., Kehr J. (2008). Identification and characterization of small RNAs from the phloem of *Brassica napus*. Plant J..

[37] Varkonyi-Gasic E., Gould N., Sandanayaka M., Sutherland P., MacDiarmid R.M. (2010). Characterisation of microRNAs from apple (*Malus domestica* ‘Royal Gala’) vascular tissue and phloem sap. BMC Plant Biol..

[38] Martin A., Adam H., Diaz-Mendoza M., Zurczak M., Gonzalez-Schain N.D., Suarez-Lopez P. (2009). Graft-transmissible induction of potato tuberization by the microRNA *miR172*. Development.

[39] Rodriguez-Celma J., Ceballos-Laita L., Grusak M.A., Abadia J., Lopez-Millan A.F. (2016). Plant fluid proteomics: Delving into the xylem sap, phloem sap and apoplastic fluid proteomes. Biochim. Biophys. Acta.

[40] Fontanili L., Lancilli C., Suzui N., Dendena B., Yin Y.G., Ferri A., Ishii S., Kawachi N., Lucchini G., Fujimaki S. (2016). Kinetic analysis of zinc/cadmium reciprocal competitions suggests a possible zn-insensitive pathway for root-to-shoot cadmium translocation in rice. Rice.

[41] Li L., He Z., Pandey G.K., Tsuchiya T., Luan S. (2002). Functional cloning and characterization of a plant efflux carrier for multidrug and heavy metal detoxification. J. Biol. Chem..

[42] Thieme C.J., Rojas-Triana M., Stecyk E., Schudoma C., Zhang W., Yang L., Minambres M., Walther D., Schulze W.X., Paz-Ares J. (2015). Endogenous Arabidopsis messenger RNAs transported to distant tissues. Nat. Plants.

[43] Ong S.S., Wickneswari R. (2012). Characterization of microRNAs expressed during secondary wall biosynthesis in *Acacia mangium*. PLoS ONE.

[44] Quan M., Wang Q., Phangthavong S., Yang X., Song Y., Du Q., Zhang D. (2016). Association studies in *Populus tomentosa* reveal the genetic interactions of *Pto-MIR156c* and its targets in wood formation. Front. Plant Sci..

[45] Ding Y., Gong S., Wang Y., Wang F., Bao H., Sun J., Cai C., Yi K., Chen Z., Zhu C. (2018). MicroRNA166 modulates cadmium tolerance and accumulation in rice. Plant Physiol..

[46] Rogers K., Chen X. (2013). Biogenesis, turnover, and mode of action of plant microRNAs. Plant Cell.

[47] Shao J.F., Xia J., Yamaji N., Shen R.F., Ma J.F. (2018). Effective reduction of cadmium accumulation in rice grain by expressing *OsHMA3* under the control of the *OsHMA2* promoter. J. Exp. Bot..

[48] Wu T.Y., Gruissem W., Bhullar N.K. (2018). Facilitated citrate-dependent iron translocation increases rice endosperm iron and zinc concentrations. Plant Sci..

[49] Cornu J.Y., Deinlein U., Horeth S., Braun M., Schmidt H., Weber M., Persson D.P., Husted S., Schjoerring J.K., Clemens S. (2015). Contrasting effects of nicotianamine synthase knockdown on zinc and nickel tolerance and accumulation in the zinc/cadmium hyperaccumulator Arabidopsis halleri. New Phytol..

[50] Bailey K.J., Leegood R.C. (2016). Nitrogen recycling from the xylem in rice leaves: Dependence upon metabolism and associated changes in xylem hydraulics. J. Exp. Bot..

[51] Nguyen C., Soulier A.J., Masson P., Bussiere S., Cornu J.Y. (2016). Accumulation of Cd, Cu and Zn in shoots of maize (*Zea mays* L.) exposed to 0.8 or 20 nM Cd during vegetative growth and the relation with xylem sap composition. Environ. Sci. Pollut. Res. Int..

[52] Axtell M.J., Meyers B.C. (2018). Revisiting criteria for plant MicroRNA annotation in the era of big data. Plant Cell.

[53] Zhang Q., Zhao C., Li M., Sun W., Liu Y., Xia H., Sun M., Li A., Li C., Zhao S. (2013). Genome-wide identification of *Thellungiella salsuginea* microRNAs with putative roles in the salt stress response. BMC Plant Biol..

[54] Meyers B.C., Axtell M.J., Bartel B., Bartel D.P., Baulcombe D., Bowman J.L., Cao X., Carrington J.C., Chen X., Green P.J. (2008). Criteria for annotation of plant MicroRNAs. Plant Cell.

[55] Wang Z., Huang J., Sun Z., Zheng B. (2015). Identification of microRNAs differentially expressed involved in male flower development. Funct. Integr. Genom..

[56] Wang Z., Huang R., Sun Z., Zhang T., Huang J. (2017). Identification and profiling of conserved and novel microRNAs involved in oil and oleic acid production during embryogenesis in *Carya cathayensis* Sarg. Funct. Integr. Genom..

[57] Yin F., Gao J., Liu M., Qin C., Zhang W., Yang A., Xia M., Zhang Z., Shen Y., Lin H. (2014). Genome-wide analysis of water-stress-responsive microRNA expression profile in tobacco roots. Funct. Integr. Genom..

[58] Liu W., Xu L., Wang Y., Shen H., Zhu X., Zhang K., Chen Y., Yu R., Limera C., Liu L. (2015). Transcriptome-wide analysis of chromium-stress responsive microRNAs to explore miRNA-mediated regulatory networks in radish (*Raphanus sativus* L.). Sci. Rep..

[59] Jin Q., Xue Z., Dong C., Wang Y., Chu L., Xu Y. (2015). Identification and characterization of microRNAs from tree peony (*Paeonia ostii*) and their response to copper stress. PLoS ONE.

[60] Li X., Shahid M.Q., Wu J., Wang L., Liu X., Lu Y. (2016). Comparative small RNA analysis of pollen development in autotetraploid and diploid rice. Int. J. Mol. Sci..

[61] Kumar R.R., Pathak H., Sharma S.K., Kala Y.K., Nirjal M.K., Singh G.P., Goswami S., Rai R.D. (2015). Novel and conserved heat-responsive microRNAs in wheat (*Triticum aestivum* L.). Funct. Integr. Genom..

[62] Dai X., Zhuang Z., Zhao P.X. (2018). psRNATarget: A plant small RNA target analysis server (2017 release). Nucleic Acids Res..

[63] Wu H.J., Ma Y.K., Chen T., Wang M., Wang X.J. (2012). PsRobot: A web-based plant small RNA meta-analysis toolbox. Nucleic Acids Res..

[64] Li Z., Zhang X., Liu X., Zhao Y., Wang B., Zhang J. (2016). miRNA alterations are important mechanism in maize adaptations to low-phosphate environments. Plant Sci..

